# Policy paper: “Nudging for better nutrition: behavioral strategies to address childhood obesity in Arab communities” policy recommendations for action

**DOI:** 10.3389/fpubh.2025.1597531

**Published:** 2026-03-09

**Authors:** Duaa Mohamed Suliman, Immanuel Azaad Moonesar, Leena A. A. Mohammed

**Affiliations:** 1 Department of Academic Affairs (Health Policy), Mohammed Bin Rashid School of Government, Dubai, United Arab Emirates; 2 School Health Physician, Sharjah Public School, Sharjah, United Arab Emirates

**Keywords:** childhood obesity, public health interventions, Arab countries, policy design, behavioral change, nudge

## Abstract

**Introduction:**

Childhood obesity is a growing public health crisis globally, particularly in Arab countries, due to changing dietary habits, sedentary lifestyles, and cultural factors. Prevention efforts focus on behavioral change strategies that promote healthy eating, physical activity, and sleep to balance energy intake and expenditure. However, the effectiveness of interventions remains uncertain, prompting ongoing research. Governments are increasingly addressing the issue through local and regional initiatives.

**Methodology:**

This research employed a scoping review approach to systematically map the available literature on behavioral strategies addressing childhood obesity, with a focus on nudging interventions within Arab communities and similar contexts, Behavioral interventions were coded using the Capability, Opportunity, Motivation–Behavior (COM-B) model to assess how existing strategies address key behavioral determinants.

**Results:**

The paper found that nudging is a soft intervention that can be easily incorporated into obesity programs and is not very costly. However, Key considerations include baseline assessments, socio-cultural aspects, multi-sectoral collaboration, and workforce capacity development in health promotion, leadership, and governance.

## Introduction

The global prevalence of overweight and obesity among children is increasing. Childhood overweight can lead to various health issues and may also negatively impact psychological well-being and social interactions. Overweight children are more likely to remain overweight into adulthood, facing persistent challenges with both physical and mental health. Additionally, childhood obesity is strongly associated with type 2 diabetes, heart disease, and an increased risk of premature mortality in middle age (1). Obesity rates are increasing on a global scale, but there is evidence indicating that young individuals in the Middle East and North Africa (MENA) region face a particularly high risk (2). A study encompassing 94 developing countries revealed that the nations with the highest prevalence of overweight were primarily situated in the Middle East, North Africa, and Latin America. Factors such as urbanization and the adoption of a modern lifestyle, combined with the wealth of oil-producing countries, have contributed to the significant surge in obesity across all age groups, particularly among children and adolescents in the region (3, 4). Many children in Arab countries consume high-calorie, nutrient-poor diets, often influenced by the increasing availability of processed foods and the cultural preference for fast food. This is compounded by limited access to physical activity in urban environments and a lack of public awareness about healthy eating habits. Despite numerous awareness campaigns and health education programs in Arab countries, progress in reducing childhood obesity has been limited. One significant reason for this is the failure to effectively change health-related behaviors among children and their families. This indicates a gap between knowledge and action, where educational efforts are not translating into sustained behavioral changes. There is growing interest in the potential of behavioral nudges to encourage dietary practices among children. This paper discusses the challenge of childhood obesity in Arab communities and explores the role of behavioral insights in improving child nutrition and reducing obesity rates in the region. Arab countries can benefit from global examples of behavioral interventions and policies that effectively address childhood obesity. These practices highlight innovative approaches that use behavioral science, community engagement, and policy tools to promote healthier habits. This paper purpose is to provide a theoretical review of policy-level childhood obesity prevention nutrition initiatives informed by behavioral insights. Exploration of behavioral insight theory applications for nutrition policies may help to inform future theoretically grounded policy-level public health interventions in the Arab region.

## Problem statement

Obesity has become a global epidemic, significantly affecting Arabic-speaking countries, particularly high-income, oil-producing nations. Among children and adolescents in these regions, the prevalence of obesity ranges from 5 to 14% in males and 3 to 18% in females. The Arab Gulf states are among the countries that have very high levels of obesity (5). In Kuwait, 30% of males and 55% of females aged 15 and older are classified as obese, giving the country the highest obesity prevalence among Arabic-speaking nations. Over the past two decades, the Arab world has experienced rapid development, leading to substantial prosperity and more convenient lifestyles. This includes advancements in transportation, reliance on affordable migrant labor, the widespread availability of Western-style fast food, and, similar to global trends, increased opportunities for sedentary living. These factors created an “obesogenic environment” around the Arabic countries (6). Furthermore, children who are overweight or obese are five times more likely to remain obese into adulthood. In addition to facing a reduced quality of life and an increased risk of premature death from related health conditions, these children often experience mental health challenges, such as bullying, low self-esteem, depression, and social isolation (7).

## Research questions

### The study will aim to answer the following

What are the key behavioral drivers contributing to childhood obesity in Arab communities?

How have behavioral interventions (“nudges”) been used globally to address childhood obesity?

What lessons can be adapted to the Arab context to influence healthier choices among children and families?

### Methodology

This research employed a scoping review approach to systematically map the available literature on behavioral strategies addressing childhood obesity, with a focus on nudging interventions within Arab communities and similar contexts. A scoping review was selected due to the exploratory nature of the topic, the diversity of intervention types, and the limited prior synthesis of behavioral insights strategies within Arab contexts. Scoping reviews are a method of knowledge synthesis that identify trends and gaps within an existent knowledge base, or scope of knowledge, for the purpose of informing research, policy, and practice. We used Arksey and O’Malley’s scoping review methodology to examine the literature.

### Search strategy

The literature search was conducted up to June 2025 to identify relevant studies, policies, and initiatives related to behavioral strategies addressing childhood obesity. A comprehensive search strategy was developed using the following databases: PubMed, Scopus, and Google Scholar. Additionally, relevant grey literature and policy documents were retrieved from authoritative organizations such as the World Health Organization (WHO), regional health bodies, and government ministries from Arab countries.

### Search terms and Boolean strategy

The following keywords and Boolean operators were used in various combinations: “childhood obesity,” “pediatric obesity,” “child overweight,” “public health interventions,” “behavioral insights,” “behavioral economics,” “nudge,” “nudging,” “choice architecture,” “action plans,” “obesity programs,” “evaluation,” “effectiveness,” “nutrition,” “diet,” “eating habits,” “healthy eating,” “Arab,” “Middle East,” “MENA,” “Gulf States,” “Saudi Arabia,” “UAE,” “Qatar,” “Kuwait,” “Jordan,” “Egypt,” “Lebanon,” “Morocco,” and “Oman.”

The search strategy combined key concepts using Boolean operators, incorporating synonyms and related terms to ensure breadth and precision. An example of the Boolean search string used was: (“childhood obesity” OR “pediatric obesity” OR “child overweight”) AND (“nudge” OR “nudging” OR “behavioral insights” OR “behavioral economics” OR “choice architecture”) AND (“nutrition” OR “diet” OR “eating habits” OR “healthy eating”) AND (“Arab” OR “Middle East” OR “MENA” OR “Gulf States” OR “Saudi Arabia” OR “UAE” OR “Qatar” OR “Kuwait” OR “Jordan” OR “Egypt” OR “Lebanon” OR “Morocco” OR “Oman”).

### Inclusion and exclusion criteria

#### Inclusion criteria

- Studies published between 2010 and June 2025.
- Publications in the English language.
- Focus on childhood obesity prevention (ages 2–18 years).
- Studies examining behavioral interventions, nudging strategies, or choice architecture approaches.
- Research conducted in Arab/MENA region or studies with clear applicability to Arab cultural contexts.
- Policy documents, systematic reviews, and grey literature from authoritative health organizations.
- Studies examining family-based, school-based, or community-based interventions.
- Research addressing dietary behaviors, physical activity, or comprehensive lifestyle Interventions.

#### Exclusion criteria

- Studies focusing exclusively on adult populations (>18 years).
- Purely clinical treatment protocols without behavioral or prevention components.
- Studies examining only pharmacological or surgical interventions.
- Research without clear behavioral change elements or nudge components.
- Studies published before 2010 or in languages other than English.
- Conference abstracts without full-text availability.
- Studies conducted in populations with significant cultural differences that limit applicability to Arab contexts
- Research focusing solely on the treatment of existing obesity without prevention elements.

### Screening and selection process

The systematic screening process followed these stages:

Stage 1: Initial Screening (Identification).

All retrieved records underwent initial title and abstract screening to assess basic relevance to childhood obesity and behavioral interventions. Records were excluded if they clearly did not meet the inclusion criteria based on population, intervention type, or study focus.

Stage 2: Full-Text Review.

Potentially relevant studies identified in Stage 1 underwent full-text review for detailed assessment against inclusion and exclusion criteria. Particular attention was paid to the presence of behavioral components, cultural relevance, and applicability to Arab contexts, and duplicates were removed.

Stage 3: Additional Sources.

Reference lists of included studies were manually searched for additional relevant publications. Expert recommendations and policy documents from Arab health ministries were also included where they met the inclusion criteria.

Stage 4: Quality Assessment.

While formal quality assessment tools were not applied, given the scoping review nature, Sources were evaluated for authoritativeness, particularly for grey literature and policy documents. Peer-reviewed publications were prioritized, with grey literature included only from recognized health organizations, government bodies, and established research institutions.

### Data extraction and analysis

A data-charting framework was developed to systematically extract variables relevant to behavioral policy design. Extracted fields included:

- Policy area/intervention type
- Country
- COM-B domain(s) addressed
- Description of key initiatives
- Nudge type (classified using the Behavioral Insights Team taxonomy)
- Implementation challenges
- Reference sources.

### Data synthesis

Due to heterogeneity in policy interventions and evidence sources, a narrative synthesis approach was applied. Data were organised thematically by policy type (e.g., school guidelines, taxation, labelling policies, digital nudges) and mapped against behavioral frameworks, including:

- The COM-B model to identify which determinants interventions targeted
- Nudge taxonomy to classify behavioral mechanisms such as defaults, salience, informational nudges, incentives, and restrictions

Cross-country patterns, strengths, and gaps were compared to identify regional trends and implementation challenges.

### Concept of behavioral economic approach and nudging?

Richard H. Thaler and Cass R. Sunstein introduced the concept of Libertarian Paternalism, which acts as the basis for Nudges, a specific behavioral intervention tool. The essence of the concept underlying this method is captured in the subsequent definition: “*Any aspect of the choice architecture that alters people’s behavior predictably without forbidding any options or significantly changing their economic incentives*.” [(8), p. 6].

Behavioral economics is an interdisciplinary field that encompasses findings from research on human behavior across disciplines such as economics, psychology, sociology, and neuroscience, offering valuable insights into how individuals make choices (behave) based on their inherent traits, life experiences, and contextual factors (7). It involves analyzing patterns of purchasing/consumption of goods (e.g., energy-dense food) in relation to constraints such as availability and price of a good and of competing goods (e.g., nutritious foods) or activities (e.g., physical activity). This field is based on the premise that individuals do not always make fully rational decisions, unlike assumptions of classical economic theory that rely on “cold” calculation of utilities, potentially improving the predictive validity (translational value) of empirical studies (9).

A nudge is a programmed change in the person’s environment to promote behavior that serves the individual’s best interest, often without their conscious awareness (10). A nudge has three primary characteristics: (1) it does not mandate individuals to adopt a specific behavior, (2) it maintains the freedom of choice, and (3) it does not provide substantial financial incentives (11).

**Figure 1:**
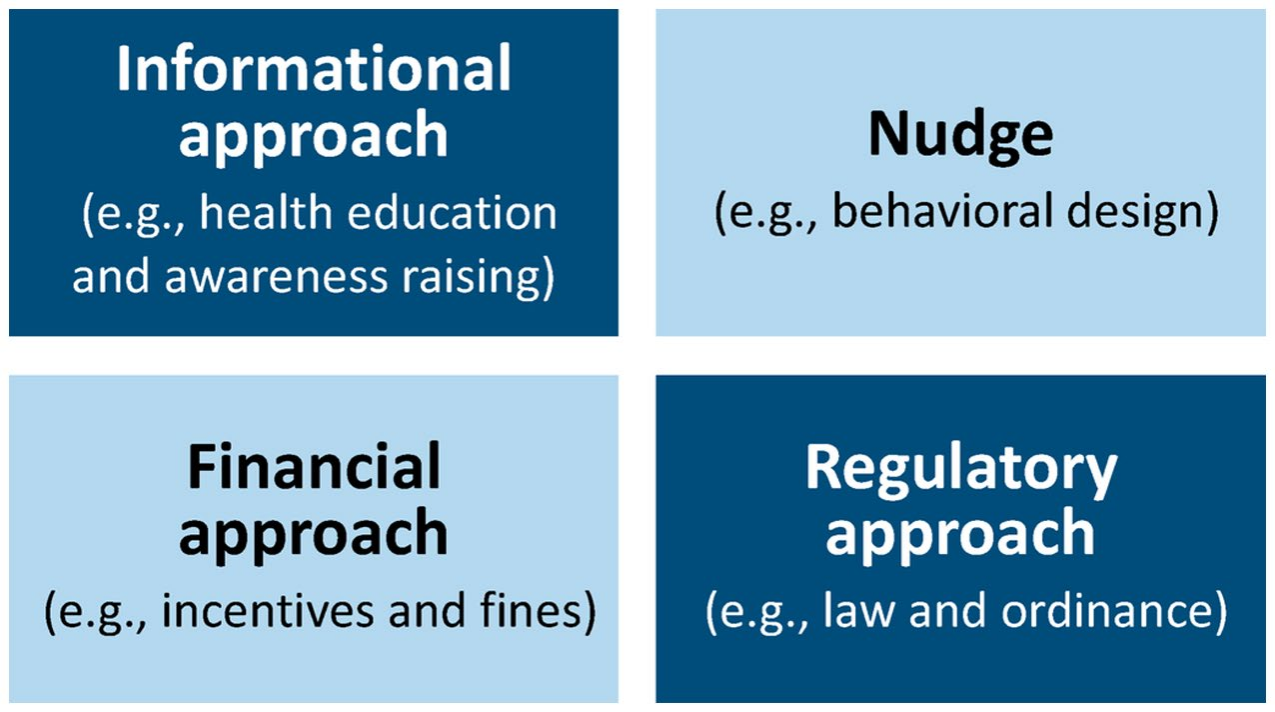
Tools for policy implementation. Adapted with permission from “Figure 1. Multiple tools for policy implementation” by Murayama et al. (11), licensed under CC BY, https://doi.org/10.3390/ijerph20053962.

Within this framework, nudge-driven policy formulation signifies a type of intervention that enhances the freedom of selection, enabling individuals to adjust their actions and make choices that advantage them. This differs from legal and economic methods, which enforce increased restrictions on the freedom to make decisions. For instance, laws like prohibiting snack items in educational institutions, banning trans fats and tobacco, or taxing unhealthy choices (such as taxes on sugar, fat, and salt). These policies require substantial regulatory oversight and include legal repercussions for noncompliance (12). Various public health domains are increasingly turning to nudging, including nutrition, where the approach can influence food choices. In general, Nudges are low-cost interventions; however, there might be indirect costs from their implementation (13). A major benefit of behavioral nudging strategies is their inexpensive execution and broad applicability across multiple societal levels, including households, educational institutions, governmental bodies, and companies. Conversely, legal and economic strategies frequently necessitate prolonged legislative procedures, rigorous enforcement systems, and increased operational expenses. Though nudges serve as effective policy instruments, they are definitely not silver bullets. Policymakers ought to view nudges as essential components in their array of policy instruments and apply them in conjunction with conventional tools like informational, financial, and regulatory strategies (Figure 1).

### Factors associated with obesity in Arab countries

The primary factors contributing to childhood obesity are largely similar between developed and developing countries. However, unhealthy dietary practices and decreased physical activity remain prominent challenges globally. A sedentary lifestyle driven by excessive engagement in indoor leisure activities, such as television watching, internet use, and computer gaming, plays a significant role in childhood obesity.

### Lack of physical activity

Several external factors further exacerbate the lack of physical activity, including limited access to open spaces and playgrounds in schools and communities. A study by Paniccià, Acito, and Grappasonni (14) emphasized that interaction with green areas, whether outdoor parks or indoor plants, is associated with reduced stress, lower hypertension and inflammation, and improved physical activity levels. Moreover, the increasing prevalence of screen time among children has raised concerns about its impact on their physical activity and overall health, as extended use of digital devices often replaces outdoor play and exercise (15). In addition, increasing academic pressures often reduce the emphasis on sports and physical education, while unsafe neighbourhoods discourage outdoor play and walking. Furthermore, some overweight children avoid participating in physical education classes or informal activities due to fear of bullying by peers (16). Collectively, these factors create an environment that fosters inactivity and contributes to rising childhood obesity rates (17).

### Excess food consumption

The process of Westernization in Arabic-speaking countries has led to numerous Western influences, particularly the increased availability of foods that are high in fats, sugar, and carbohydrates (6). Furthermore, unrestricted access to energy-dense, fast foods in school cafeterias, vending machines, and nearby outlets significantly contributes to excessive caloric consumption among schoolchildren. This issue is often exacerbated by limited knowledge about proper dietary components among students. Additionally, the practice of overfeeding low-birth-weight infants to promote catch-up growth, if prolonged, can increase the risk of obesity later in life (17).

### Sociocultural factors and urbanization

Supportive people can significantly impact behavior change, either facilitating or obstructing it, while social circles can affect weight loss outcomes. For children, Parental engagement is an essential element of successful obesity treatment programs (18). However, this engagement is often limited when parents fail to recognize or acknowledge their child’s overweight status. Research has indicated that most parents do not correctly understand their child’s weight status (19).

Parental practices also contribute to children’s obesity. Overprotective behaviors, forced feeding, combined with traditional misconceptions about health and nutrition, as well as limited nutritional knowledge among parents and caregivers, contribute to childhood obesity. Additionally, the educational background of parents significantly influences their children’s diet. For example, a study in Italy employing the KIDMED index found that greater parental education, not income or housing situation, was notably associated with better adherence to the Mediterranean Diet (20).

Beyond the family, Peers’ influence can act as a vital source of encouragement for children and teenagers trying to lose weight. Evidence of how peers and friends affect the health behaviors of children and adolescents found that engaging peer networks in prevention and intervention initiatives effectively changes health behaviors (18).

### Religious and cultural influence

Cultural factors may exacerbate the obesity issue. For instance, in Saudi Arabia and Kuwait, social gatherings often involve increased food consumption, with traditional meals typically consisting of rice (high in carbohydrates) and meat (high in fat), which are shared among large groups.

Ramadan and Religious Observances: The Islamic holy month of Ramadan presents unique challenges and opportunities for nutrition interventions. Fasting patterns significantly alter daily eating habits, with concentrated food consumption occurring during Iftar (evening meal breaking the fast) and Suhur (pre-dawn meal). Post-Iftar behaviors often involve increased social eating and consumption of traditional sweets and fried foods. Tailored interventions during Ramadan can include pre-Ramadan habit formation campaigns, balanced Iftar meal promotion, and health messaging embedded within religious contexts that emphasize moderation and balance—core Islamic principles of well-being.

### Gender dynamics and food decision-making

Maternal vs. Paternal Influence: In Arab families, mothers typically bear primary responsibility for meal planning and preparation, while fathers often make decisions regarding household food budgeting. As a result, Interventions must address both decision-making levels, with messaging targeted appropriately to each gender’s sphere of influence. Moreover, Children in Saudi Arabia whose fathers were overweight or obese were nearly twice as likely to be overweight or obese themselves compared to those whose fathers had a normal weight (21).

Cultural Body Image Expectations: Boys and girls face different cultural pressures regarding body size and eating behaviors. Traditional preferences for larger body sizes in some Arab cultures, particularly for young children, may conflict with obesity prevention messages. Moreover, research exploring the relationship between gender and body image has shown that males tend to report greater satisfaction with their bodies compared to females, despite generally having a larger body size. Therefore, gender-specific interventions should address these cultural norms while promoting health rather than appearance-focused goals (22).

### How behavioral economics can help overcome obesity in children

Nudges can be utilized across multiple health policy areas and organizational functions, such as preventive healthcare, health and non-health service delivery, long-term care/dementia prevention, community-based care frameworks, and technological advancements. The World Health Organization (WHO) projected that by funding the most efficient and practical measures to prevent and manage NCDs in low- and middle-income nations, a seven-fold return might be realized by 2030 (11).

Family-based behavioral weight loss programs (FBT) targeting childhood obesity frequently incorporate incentive systems centred around positive reinforcement. Insights from behavioral choice theory and behavioral economics have informed the development of obesity treatments and how to tackle this issue. For Example:

- Enhancing the reinforcing value of alternatives, increasing the appeal of healthier or more rewarding alternatives
- Raising the behavioral cost associated with acquiring unhealthy foods (i.e., making it harder to obtain food).
- Stimulus control, altering the surroundings to enhance the chances of participating in preferred behaviors. In obesity treatment, individuals are motivated to boost the presence of prompts for healthy actions in their surroundings (e.g., placing a fruit bowl on the counter, ensuring running shoes are visible) and to reduce the presence of prompts for unhealthy actions (e.g., removing unhealthy food options from the home, relocating the television from the living area) (18).

In many cultures, obesity is often stigmatized as a personal flaw, despite advances in understanding its complex causes, including genetic, cultural, and environmental factors. This bias is also evident in the medical community, where a blaming approach can harm the therapeutic relationship and create barriers to behavioral change, such as low self-esteem and hopelessness. Families affected by obesity are often sensitive to the topic, making it crucial to avoid blame and instead focus on addressing modifiable environmental risk factors (17).

Despite significant efforts to promote healthy eating and increased physical activity, notable improvements in these behaviors or obesity outcomes remain limited. The reason lies in the gap between evidence-based obesity guidelines and their translation into behaviorally informed, patient-centred recommendations. To combat the obesogenic environment and reduce the global prevalence of childhood obesity, a multifaceted policy approach is essential. Policies guided by ‘behavioral insights’ (BIs) have shown promise in enhancing children’s dietary habits. While, behavioral economics provides a framework for how we counsel patients through small changes (23).

When applying nudges, considering social and cultural contexts is essential. Reports indicate that both the acceptance of nudges by individuals and the effectiveness of nudges varied across different countries and regions. This indicates that the applicability of the nudge method requires additional examination, and policymakers need to interpret the outcomes of nudges from various social or cultural backgrounds with caution (11).

### Theoretical framework

#### Behavioral analysis using the COM-B model

**Figure 2:**
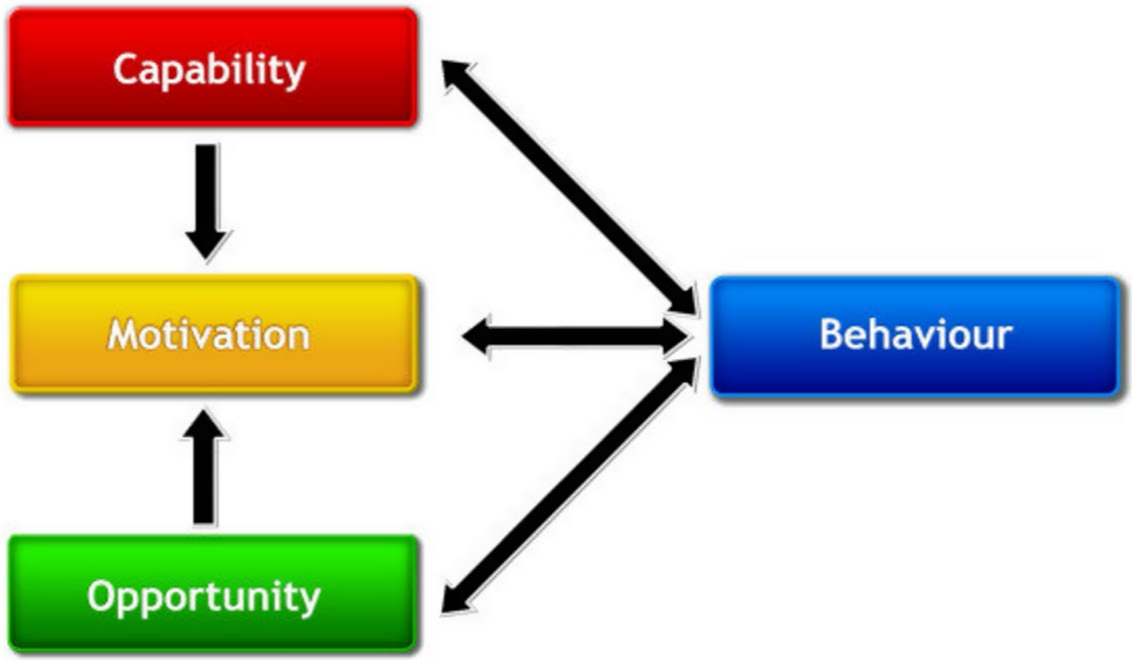
Conceptual representation of the COM-B model of behaviour change. Adapted with permission from Michie et al. (24), https://www.behaviourchangewheel.com/.

The behavior change wheel serves as a framework for creating and assessing interventions. At the centre of the behavior change wheel is the COM-B model of behavior, representing Capability (C), Opportunity (O), Motivation (M), and Behavior (B). This model suggests that the three components impact behavior, encompassing all external factors that enable the behavior to occur. The model suggests that both Capability and Opportunity impact Motivation, positioning it as the key mediator; thus, Capability and Opportunity influence behavior directly and indirectly. As per the COM-B model, changing behavior requires altering one or more of its components, which pertain to the behavior itself or related supportive or competing behaviors (24). In this study the COM-B model is used to assess the three core components necessary for a behavior to occur—Capability, Opportunity, and Motivation—and how they interact to influence childhood nutrition behaviors in Arab communities (See Figure 2).

#### Global examples of nudge practice: focusing on childhood obesity

Globally, behavioral interventions or “nudges,” have been effectively used to shape healthier eating behaviors among children by altering their choice environments rather than relying solely on education. The majority of scholarly evidence regarding nudge interventions has been obtained from Western nations, yet there is a notable collection of instances of nudge methods in non-Western countries, including those in the Western Pacific region (11). This section presents case studies demonstrating the application of behavioral strategies in promoting healthier eating behaviors in Children.

#### 1-health choice default nudge

Walt Disney World, USA (25): Research examined children’s meal choices at Walt Disney World after the implementation of healthier default sides (such as grapes, apple slices, unsweetened apple sauce, or baby carrots) and drinks (low-fat milk, water, or 100% juice) instead of the former defaults of fries and a soda. Nutrient-rich default sides were shown on visual displays, but the entire selection was detailed on the menu, allowing children to choose not to have the healthy default in Favor of fries or a soda. Sales information was gathered from 94 quick-service establishments and 51 table-service establishments during a 3-year period after the introduction of healthful defaults, but pre-intervention data could not be obtained since the healthful sides were not offered before they were set as the default option. Children, on average, accepted the defaults for quick-service restaurants (49.4% for sides, 67.8% for beverages) more frequently than at table-service restaurants (40.3% for sides, 45.6% for beverages), indicating that children and their caregivers might be more amenable to default healthful choices.

Behavioral Marker: Proportion of children accepting healthy defaults.

COM-B Link: Increases Opportunity by simplifying food choices; leverages Motivation via social norms.

Arab Context Consideration: Could be adapted to school and restaurant meals in Gulf countries, incorporating culturally appropriate foods and beverages.

Policy Example: In consideration of reducing decision-making between healthful and less healthful options, in September 2018, California passed the “Healthy By Default” Kids’ Meal Beverages Bill, which requires either milk or water to be the default beverage for children’s meals in restaurants (Voices for Healthy Kids Action Center, 2019). This default strategy ties directly to BE theory, as children tend to seek the easiest option and accept the norm, reducing their decision-making burdens by accepting what is offered to them (26).

#### Case study of healthy incentives program

The Healthy Incentives Pilot (HIP) was a U. S. Department of Agriculture initiative involving 5,000 low-income households that received 30 cents back for every SNAP dollar spent on select fruits and vegetables (TFVs). These included fresh, frozen, canned, or dried produce without added sugars, fats, or salt. The program ran from 2011 to 2012 and led to a 26% increase in TFV consumption among participants. While the results suggest that financial incentives can effectively encourage healthier food choices, concerns remain about whether these behaviors are sustained once incentives are removed (27).

- Behavioral Marker: Change in household fruit and vegetable purchasing.
- COM-B Link: Enhances Motivation by increasing the perceived reward for healthier choices.
- Arab Context Consideration: Similar programs could be implemented in subsidized school meal schemes or community programs, with attention to cultural preferences in fruit and vegetable selection.

#### Sugary drink tax

The tax on sugary beverages has gained significant backing and debate in recent times. Behavioral economics theory suggests that imposing taxes on sugary beverages would lower their demand, leading to a decrease in consumer purchases. According to Petrescu et al. (10), nudge interventions to reduce consumption of sugar-sweetened beverages seem similarly acceptable in the UK and USA, being more acceptable than taxation. The results of this review and meta-analysis demonstrate that Nudge Theory strategies provide an effective and viable public health strategy in encouraging healthier eating choices in adults (28).

A study of the sugary drink tax in Mexico indicated a 6% reduction in the buying of taxed drinks and a 4% rise in the purchase of other drinks like water during the initial year of enforcement (53).

- Behavioral Marker: Volume of taxed sugary drinks purchased or consumed.
- COM-B Link: Modifies Opportunity by changing the environment and Motivation via cost disincentives.
- Arab Context Consideration: Many Gulf countries have begun implementing soft drink taxes, offering a contextually relevant policy leverage point.

#### Advertising regulations

In 2014, the Mexican government introduced a policy measure aimed at addressing and reducing its obesity prevalence through limiting television ads for high-calorie foods and soft drinks during weekdays between 14:30 and 19:30, and on weekends from 07:30 to 19:30 (29).

- Behavioral Marker: Exposure of children to unhealthy food advertising.
- COM-B Link: Alters Opportunity by reducing environmental cues for unhealthy consumption.
- Arab Context Consideration: Similar regulations could reduce exposure to energy-dense foods in children’s media programming, particularly during Ramadan or school holidays.

#### Choice architecture/environmental restructuring

A notable example of applying behavioral insights to tackle childhood obesity is the UNICEF and Behavioral Insights Team (BIT) collaboration in North Macedonia. The intervention focused on reshaping the food environment in and around schools, recognizing that children’s choices were heavily influenced by the visibility and accessibility of unhealthy food options. Rather than relying solely on health education, the initiative emphasized changing the “choice architecture,” for instance, promoting healthier options in school kiosks and improving food labelling. Social and cultural factors were also addressed, including parental practices of giving children money as a means of social bonding, which often led to unhealthy food purchases, and the common misperception that traditional foods are inherently healthy (Bianchi, 2021).

- Behavioral Marker: Proportion of healthier food purchased in school kiosks; adherence to school nutrition guidelines.
- COM-B Link: Opportunity enhanced by altering physical and social environments.
- Arab Context Consideration: Could be adapted to Arab schools, considering parental involvement, religious observances, and food affordability.

In Korea, the findings of a study targeting the child obesity management program utilizing the nudge technique and inducing autonomous weight management through environmental control have not only enhanced the height, obesity, and BMI of overweight children but also fostered better exercise habits and knowledge about obesity (30).

- Behavioral Marker: Changes in BMI, exercise frequency, knowledge of obesity.
- COM-B Link: Enhances Capability (knowledge) and Motivation (autonomous goal setting).

#### Salience and positioning nudge

In New Mexico, USA, a simple change in the design of a shopping trolley resulted in people making better decisions about the food they purchase. Researchers marked a line with yellow duct tape across the width of the trolley and added a sign asking shoppers to place fruit and vegetables in front of the line and everything else behind it. The result was a 102% increase in sales of fruit & vegetables (at no loss of profitability to the retailer) (31).

- COM-B Link: Increases Opportunity by making healthier options more visible and accessible.
- Arab Context Consideration: Positioning and salience strategies could be applied in grocery stores, school cafeterias, and food courts, culturally adapted to local produce and traditional meals.

## Findings and analysis

### Behavioral economic application to policy-level childhood obesity prevention in the Arab region

To contextualize global behavioral strategies within Arab settings, we reviewed ongoing interventions across Arabic-speaking countries and categorized them by behavioral strategy, implementation challenges, and alignment with COM-B frameworks. Table 1 presents a summary of this mapping exercise, highlighting where behavioral components have been effectively used or underutilized. (Table 1) It outlines key initiatives aimed at promoting healthier lifestyles, such as mandatory guidelines for school meals, nutritional awareness campaigns, front-of-pack labelling, and taxation on unhealthy foods. A range of intervention strategies & nudges were employed across the Arabic countries, with some studies detailing multiple, combined behavior change strategies (including nudges), others incorporating few strategies.

**Table 1:** Current policies on nutrition and childhood obesity in Arab countries [summary of literature search (N = 15)].

Policy area/Intervention	Country	COM-B domain(s)	Key initiatives	Behavioral component (Nudge)	Challenges	References
Mandatory Guidelines for School Meals	UAE, KSA Kuwait	Opportunity, Capability	Regulates school cafeterias to provide balanced meals, reduce sugary snacks, and promote water consumption.	Choice restriction Default, Choice Architecture	Ensuring compliance and consistent implementation across schools.	(32, 35, 49–51)
Public Health Campaigns	Qatar, KSA, UAE	Capability, Motivation	Programs like Kuwait’s Healthy School Initiative educate students on nutrition and active lifestyles.	Informational nudges	Limited parental involvement and challenges in sustained engagement	(46, 52) (WCM-Q, 2021)
Implementation of Front-of-Pack Nutrition Labelling	GCC countries (e.g., UAE, Saudi Arabia, Oman), Morocco	Capability	Front-of-pack labelling provides clear information about sugar, salt, and fat content. The nutrient-specific traffic light labelling (Islamic Republic of Iran, Kingdom of Saudi Arabia, United Arab Emirates);	Salience and heuristics	There are currently three types of front-of-pack labels in use or under development in the Eastern Mediterranean Region. Namely, traffic lights systems, Nutri-Score, and health logos. Variability in consumer understanding and adherence to guidelines by food manufacturers.	(33, 36)
Taxation on Unhealthy Foods	Saudi Arabia, UAE, Oman	Motivation	Excise taxes on sugary beverages (50–100%) aim to reduce consumption.	Incentives (Financial Disincentive)	High costs of alternatives and potential resistance from the food industry.	(44)
Subsidies for Healthy Foods	Egypt, Morocco	Opportunity	Subsidizes staple foods like fruits and vegetables to improve affordability.	Incentives (Positive), Price Framing	Ensuring accessibility for low-income families and maintaining quality of subsidized items.	(43)
Advertising Regulations for Children’s Media	KSA, Lebanon, Jordan, Oman, UAE, Qatar	Opportunity	Restricts unhealthy food advertising during children’s TV programming and social media.	Restrictive Cue	Weak enforcement mechanisms	(33, 41, 42)
Intensive weight-loss camp	Qatar	Capability, Motivation	Builds structured routines, increases physical capability, enhances motivation through group support and nudges	social norm nudges	Highly resource-intensive; limited scalability; short-term changes risk relapse	(46)
Digital and Mobile Nudges (SMS, Apps)	UAE	Motivation, Opportunity	Provide timely prompts to encourage healthy eating habits and track progress.	Timely Prompts, Feedback, Gamification	Limited use in public programs; access and digital literacy issues in lower-income populations.	(15)

### School-based nutrition programs

Arab governments have introduced initiatives to promote healthier food options in schools: Mandatory Guidelines for School Meals: Countries like the UAE and Saudi Arabia regulate school cafeterias to provide balanced meals, reduce sugary snacks, certain food items (e.g., alcohol, carbonated drinks, pork, and allergens) are prohibited and promote water consumption. According to the School Healthy Eating and Food Safety Policy in UAE, Schools must foster a nutritious food environment that promotes a nutritious eating atmosphere filled with healthy foods for every individual in the school (32). Schools must guarantee that the meals provided are healthy, focusing on lowering excessive amounts of sugar, salt, and fats. Sustainable and secure food methods are crucial, spanning from production to consumption. Furthermore, the MASAR project, in the UAE, is implementing a set of interventions is being implemented to reduce childhood obesity in schools by raising health awareness among students and their families, improving school food quality, and providing opportunities for safe physical activity. Additionally, the project offers behavioral intervention programs to students and their parents who are experiencing weight issues (33). According to Dubai’s 2024 Food and Nutrition Guidelines are transforming meals and nutrition in educational institutions from nurseries to universities. The revised guidelines emphasize healthier food choices, sustainability (such as plant-based dishes and environmentally friendly packaging), and involving students with technology through initiatives like the “Food Heroes Plate” and “SCAN the Block” for food education. Schools also implement “healthy eating points” to encourage nutritious choices (15).

In 2004, the Ministry of Health and Education in Saudi Arabia established the “Regulations of Health Conditions for School Canteens,” which has been compulsory for implementation since 2014. The guidelines feature a compilation of canteen food items that are either permitted or prohibited (34). In Kuwait, authorities introduced a new policy for school cafeterias with the goal of encouraging healthy eating practices and lowering student obesity (35).

### Food labelling and advertising restrictions

Nutritional Labelling: The GCC countries have adopted front-of-pack labelling (traffic light systems) to provide consumers with clear information about sugar, salt, and fat content.

In Morocco, to lower the sugar content of processed food products the country has implemented a sugar tax and the Nutri-Score front-of-pack labelling scheme (33). As per this GCC-wide standard, prepackaged Food product labels must be in Arabic or contain a translation of the label in Arabic. Producers and retailers are required to offer a list of the nutrient content for pre-packaged food items, even in the lack of a health or nutrition assertion (36–38).

In Dubai, the Food and Nutrition Guidelines for 2024 require all meals provided in educational settings, including buffet and grab-and-go selections, to have food labelling. The regulations indicate that labels should contain nutritional details according to the serving size, which represents the actual portion eaten by students, instead of per 100 grams. Moreover, allergen details, including prevalent allergens like nuts, dairy, gluten, and more, should be prominently shown to protect students with food sensitivities (39). These labels should be available in both Arabic and English to guarantee clarity and accessibility for all parties involved, such as students, parents, and school personnel (15, 40). The SEHHI Healthy Menus initiative displays the SEHHI logo on healthy menus in restaurants and food establishments. Foods featuring the SEHHI logo contain less fat, reduced sugar and salt levels, and increased fiber. The initiative is part of the broader SEHHI programme that is led by the Abu Dhabi Public Health Centre (36).

### Advertising regulations

A review indicated that legislative measures to control food marketing aimed at children are quite scarce in the Middle East region (41). Advertising regulations pose a number of challenges, including restricting marketing to children (particularly in relation to cross-border marketing, digital marketing, and monitoring of marketing) (33). In countries like Lebanon and Jordan, advertising unhealthy foods during children’s TV programming or on social media is restricted, although enforcement varies. In Kuwait, many recommendations have been adopted to limit the inappropriate marketing of complementary foods up to 36 months. The legislation prohibits the promotion of complementary foods for infants younger than 6 months, as well as the promotion of complementary foods on packaging for complementary foods (42).

### Subsidies for healthy foods

The Farm-To-School Program (F2SP) is a competitive grant initiative aimed at participants of the National School Lunch Program and School Breakfast Program, focusing on enhancing the amount and regularity of locally sourced fruits and vegetables provided to students throughout the academic year. The World Food Programme (WFP) is at the forefront of humanitarian initiatives. Egypt and Morocco have both gained advantages from their assistance and have observed enhancements in the number of children receiving lunches at school. Egypt has established a continuous centralized school nutrition initiative in collaboration with the World Food Programme (43).

### Taxation on unhealthy foods

Sugary Drink Taxes: Saudi Arabia, the UAE, and Oman have implemented excise taxes on sugary beverages, with rates up to 50–100%, aimed at reducing consumption. Implementing a sugar tax on sugary drinks has led to large reductions in the consumption of sugar from sugary drinks in the UK, primarily through manufacturers’ reformulation of sugary drinks without affecting sales (16). Sugar taxes were first implemented in GCC countries in 2017. Saudi Arabia and the UAE currently charge 50 to 100% on most sweetened drinks. Sugar taxes, however, have a limited reach and scope in the region. Moreover, governments can use the revenues from these taxes to support health and fitness programs (44).

### Public health campaigns

Qatar’s Your Health First (YHF) is a national, multi-faceted health campaign designed to change unhealthy behaviors into healthy ones. By educating the population about health, obesity and diabetes. The campaign collaborated with the Ministry of Education and Higher Education for the second consecutive year to make sure children returned to school aware of the significance of health. The camping provided children with school bags, lunchboxes, water bottles, and activity books in preparation for their first day returning to school after the extended summer break (45). The Ministry of Health and the Ministry of Education have initiated a public health awareness campaign for childhood obesity in Saudi Arabia called the “RASHAKA Initiative.” This program seeks to encourage healthy habits in students by enhancing eating practices, boosting exercise levels, and educating them about the dangers of obesity (46).

### Intensive weight loss camp

In Qatar, a study evaluated a three-year integrated intervention (Agder) targeting overweight and obese school children aged 9–12. In the first year, 941 children from four intervention schools were screened, and 430 (45.7%) were found to be overweight/obese. One hundred children with BMI ≥ 95th percentile completed a two-phase program: (1) an 11-day intensive weight-loss camp combining physical activity, dietary control, lifestyle education, and behavioral nudges; followed by (2) 10 weeks of after-school clubs focused on weight maintenance, motivation, and parent engagement. The camp led to a significant 12.5% reduction in BMI SDS (*p* < 0.001) for all participants, with girls showing slightly greater improvements than boys (46) (Table 2).

**Table 2:** Three-level behavioral nudge framework for childhood obesity prevention in Arab communities.

Target level	Key barriers	COM-B Domain(s)	Nudge type	Behavioral/policy counter-strategies
Parents	- Limited nutrition knowledge- Preference for convenience/processed foods- Influence of extended family on food choices- Financial constraints	Psychological Capability, Motivation, Social Opportunity	Reminders, Pre-commitment, Incentives	- SMS/app reminders for healthy meal planning- Family pre-commitment pledges- Subsidies or vouchers for fruits and vegetables- Culturally adapted nutrition workshops- Inclusion of extended family in educational campaigns
Schools	- Unhealthy cafeteria options- Limited infrastructure for physical activity- Low awareness among teachers/staff- Peer influence on unhealthy eating	Physical Opportunity, Social Opportunity, Psychological Capability	Defaults, Priming, Social Norms	- Default healthy meals and portion control in cafeterias- Visual priming via culturally relevant posters/signage- Structured daily physical activity sessions- Teacher training on nutrition and behavioral strategies- Peer ambassador programs to reinforce healthy norms
Children	- Preference for sugary/high-fat foods- Exposure to junk food marketing- Peer pressure around food choices- Limited autonomy in choosing healthy foods	Reflective & Automatic Motivation, Psychological Capability	Salience, Incentives, Proximity, Digital, Social Norms	- Gamified apps and virtual challenges promoting fruit/vegetable intake- Front-of-pack traffic light labels for unhealthy foods- Placement/proximity nudges in vending machines and school stores- Social norm campaigns using peers and local influencers- Rewards and recognition for healthy choices

### Involvement of technology

Dubai 2024 Food and Nutrition Guidelines incorporate technology to enhance the engagement of nutrition education. The “Food Heroes Plate” aids students in grasping balanced meal sizes, while online resources allow parents to monitor school meals, enhancing communication between home and school. Moreover, food labeling is essential in directing healthier food selections (15).

### Gaps in policy implementation and Behavioral interventions in Arab countries

While the existing policy landscape in the Arab region reveals a growing commitment to addressing childhood obesity, these interventions often lack a cohesive behavioral foundation and struggle with sustained impact. Most programs remain education-heavy, increasing knowledge but failing to shift daily habits or automatic behaviors. Even intensive interventions, such as the Qatar weight-loss camp, show short-term improvements but struggle with long-term maintenance, largely because children return to environments that lack supportive and reinforced habits.

Policies are an integral part of health promotion, and certain policies related to school environments, food labelling, and others are crucial in addressing childhood obesity (5). When seeking to reduce childhood obesity, national governments have often adopted similar top-down initiatives such as Higher taxes on sweet beverages, National dietary and physical activity guidelines, School food regulations, and Restrictions on marketing (47). There are a number of possible explanations for government action not yet achieving a reduction in childhood obesity in most countries (47). Arab countries lack important local policies in schools, such as healthy food and drinks in school canteens, regular screenings for overweight and obesity, and extracurricular activities (5). One of the major gaps in policy is weak Policy Enforcement; policies to promote the implementation of simplified nutrition information are limited in the MENA region (48).

### Limited nudges targeting caregivers and home environments

The majority of initiatives in Arab nations have centred around schools or campaigns, mainly targeting children and teenagers. Nonetheless, children’s eating habits are greatly influenced by family interactions and household settings. There is a significant absence of nudging techniques targeting parents and caregivers, who play essential roles in decisions regarding grocery purchases, meal cooking, and screen usage. Interventions offering gentle prompts for parents, like serving size labels on food packages, fridge magnets featuring dietary visuals, or text messages reminding caregivers to add fruit to lunchboxes, are still uncommon. Additional family-focused strategies are essential to cultivate healthier food environments at home and support initiatives in schools. To create a family-oriented incentive system, parents and children collaborate to identify suitable and attractive rewards for achieving goals. Optimal incentives are those that enhance social support and strengthen the importance of specific activities, like a family biking trip or a stroll with friends.

There is limited contextualization of behavioral interventions, reducing their acceptability and sustainability in Arab cultural settings. Many strategies are borrowed from Western contexts without adapting them to local food preferences, religious norms (e.g., fasting periods), or societal structures. These gaps underscore the need for a comprehensive, culturally grounded behavioral framework.

### Limited use of digital nudges

While digital engagement is high across Arab countries, mobile apps and social media are underutilized as tools for nudging healthy behavior in children and families. Digital tools present a vast, underutilized opportunity for scaling nudges across the MENA region, especially among tech-savvy youth. Apps and platforms could be used to send real-time reminders, gamify healthy behaviors, or customize meal suggestions. For example, apps that nudge users to drink water before meals, reward daily step goals, or highlight the healthiest nearby food options are widely used in high-income settings but are almost absent in regional public health strategies. Governments and local innovators can collaborate to develop culturally appropriate, behaviorally designed digital solutions that are accessible across income levels.

### Defaults are underutilized

The majority of initiatives in the Arab region depend on education and regulation instead of utilizing behavioral insights such as nudges or incentives. Behavioral science shows that defaults, pre-chosen options requiring no active decision-making, are among the most effective nudges for encouraging healthier choices. Within Arab nations, however, default mechanisms remain significantly underused. For instance, school cafeterias and food venues could automatically provide healthy meals (such as fruits, vegetables, and water) unless students opt to refuse. Nevertheless, most school food policies focus on restrictions or rules rather than fostering environments where choosing healthy foods is the simplest choice. Enhancing the use of defaults, such as placing healthy options at eye level, incorporating pre-packaged fruit in meal sets, or designating healthier combinations as the norm in digital food ordering platforms, can subtly shift consumption patterns while preserving the freedom to choose.

### Salience and emotional appeal drive engagement

Campaigns that employ compelling visuals, emotionally resonant messages, and trustworthy spokespeople typically perform better than those that focus only on providing information. When children and families come across campaigns associating health behaviors with compelling visuals (such as sugar turning into fat in the bloodstream), captivating stories, or endorsement from well-regarded figures like local influencers, clergy, or renowned athletes, the message is likely to enhance motivation and memory retention. In many Arab countries, these approaches have only recently begun to be integrated into health promotion strategies. Integrating these behavioral tactics into public educational initiatives—especially through social media channels—can greatly enhance message retention and behavior adoption.

### Affordability remains a structural barrier

Although behavioral nudges are essential, they need to be integrated into supportive structural environments. In low- and middle-income areas throughout the MENA region, cost is a significant limitation. Nutritious choices frequently come at a premium compared to processed options, and despite being aware, families might still opt for less healthy food because of expenses. Taxes on sugary beverages have advanced in curbing unhealthy intake, but beneficial incentives like subsidies for fruits, vegetables, and whole grains are less frequent and less focused. Pricing strategies informed by behavior (e.g., loyalty rewards for nutritious purchases, cash-back offers, mobile discounts) can close this affordability gap and encourage sustainable decisions. In low-income countries such as Yemen and Sudan, healthy food options remain unaffordable and inaccessible for large portions of children.

### Scarcity of robust behavioral impact evaluations

Despite the adoption of various health campaigns and policy reforms in the region, rigorous behavioral evaluations are lacking. Most monitoring focuses on health outcomes (e.g., BMI, diabetes risk) rather than the specific behavioral changes leading to them (e.g., sugar reduction per week, snack replacement habits, label-reading frequency). Without clear behavioral metrics, it is difficult to assess the real efficacy of nudges or modify them based on real-world results. Building local research capacity to design randomized controlled trials, A/B testing of messages, and pre-post behavioral assessments is critical for scaling evidence-based interventions.

This gap analysis underscores the need for Arab countries to transition from traditional health promotion methods toward systematically embedding behavioral science principles into policy design and implementation. Closing these gaps will require stronger enforcement, cross-sectoral coordination, digital innovation, and a deeper understanding of local behavioral drivers. To bridge this gap, there is a need to move beyond traditional approaches and structural policies toward strategies informed by behavioral science. The following section explores global and regional evidence on the effectiveness of such behavioral interventions. By analyzing successful case studies and identifying context-specific barriers and facilitators, we aim to extract actionable insights that can inform a more robust and adaptive policy approach in Arab countries.

### Evidence-based policy approaches to promote healthy weight among children

Behavioral strategies are increasingly being used in health promotion due to their potential to subtly guide people toward better choices without coercion. In the context of childhood obesity in Arab communities, these strategies can complement existing policies by closing the gap between awareness and action.

### Policy design framework for childhood obesity prevention in Arab communities

Addressing childhood obesity in Arab communities requires more than isolated interventions; it demands a comprehensive, multi-level approach that considers the interplay of individual behavior, social environments, and cultural norms. Behavioral strategies, particularly nudges, offer a subtle yet powerful mechanism to guide healthier choices without coercion. By targeting parents, schools, and children, policies can influence decision-making at every stage of a child’s daily life, from household food purchases to school meals and personal eating habits.

The Three-Level Behavioral Nudge Framework presented here integrates evidence from regional studies, global behavioral science, and practical case studies from the Arab world. It identifies common barriers at each level and proposes contextually adapted counter-strategies, ensuring interventions are both effective and culturally sensitive. By mapping barriers, COM-B domains, and nudge types, this framework provides actionable guidance for policymakers, educators, and community stakeholders aiming to create supportive environments for healthy behavior.

### The childhood obesity prevention policy aims to influence the behavior of three primary target groups

1. Parents: As the heads of households, parents control household spending and make key decisions about food purchases. Their choices are pivotal in determining their children’s access to nutritious food and shaping their dietary habits.
2. Schools: Since children spend a significant portion of their day in school, often consuming breakfast and lunch there, schools have a vital role in fostering healthy eating habits. A supportive school environment can encourage positive dietary behaviors.
3. Children: Ultimately, children make the final decision about the food they consume. Establishing healthy eating habits during childhood is crucial, as these behaviors often persist into adulthood.

### The proposed Arab context nudge model (ACNM) for the prevention of childhood obesity

Building on the evidence-based policy framework outlined above and strengthening the effectiveness of childhood obesity interventions in the region, this paper proposes the Arab Context Nudge Model, a culturally grounded behavioral framework that integrates the COM-B system with cultural components. The proposed Arab Context Nudge Model (ACNM) serves as a culturally grounded behavioral framework designed to systematically enhance childhood obesity interventions in the Arab region.

1. Purpose and Rationale

**Figure 3:**
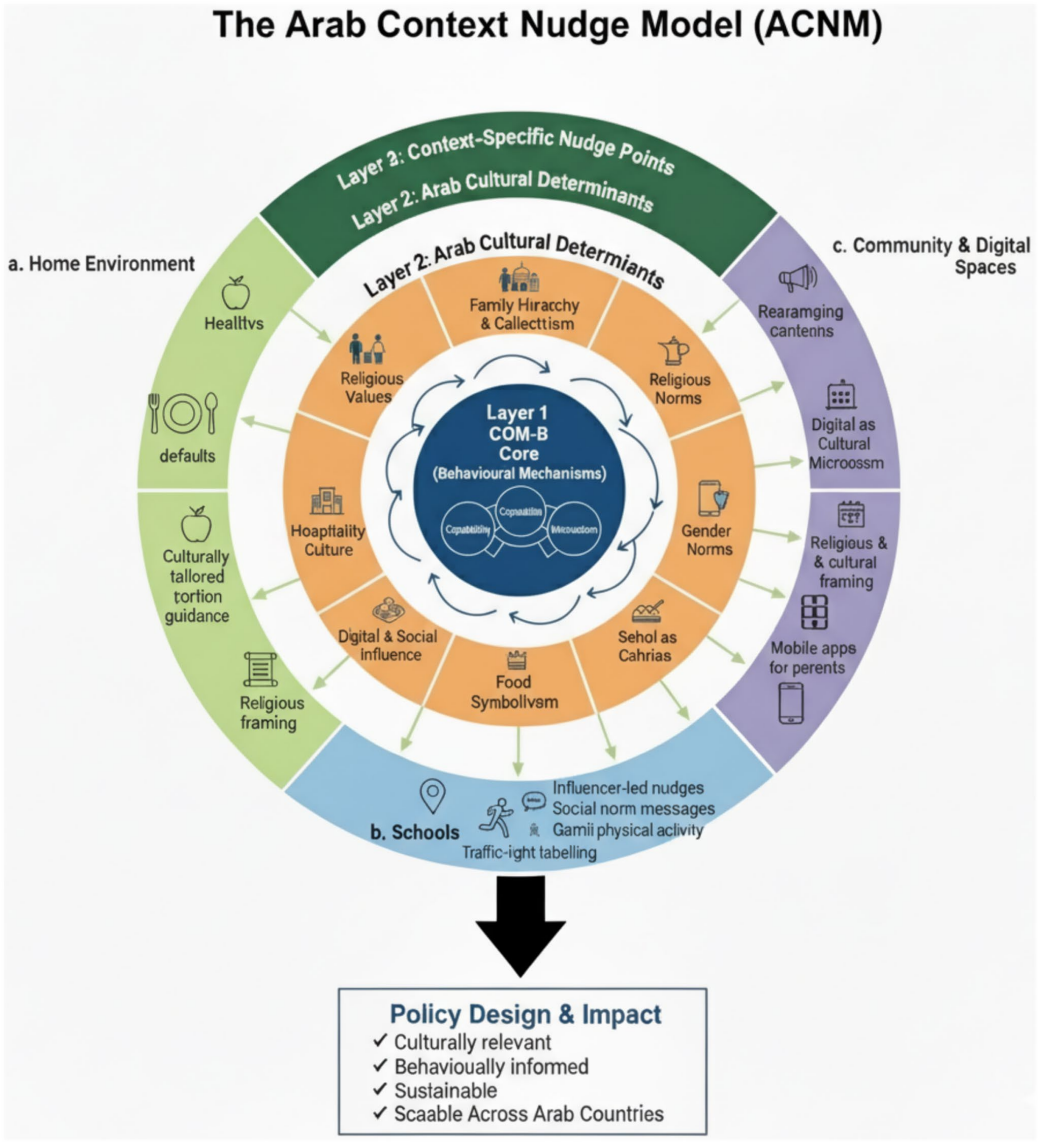
The proposed Arab context nudge model (ACNM).

Structurally, the ACNM integrates three key layers: Layer 1: The COM-B Core (Capability, Opportunity, Motivation) identifies the fundamental behavioral mechanisms; Layer 2: Arab Cultural Determinants specifies region-specific factors like Family Hierarchy, Hospitality Culture, and Religious Values that modify the COM-B drivers; and Layer 3: Context-Specific Nudge Points defines the realistic settings—Home, Schools, and Community/Digital spaces, where culturally tailored nudges can be applied. By explicitly linking behavioral science with local norms and environments, the ACNM provides policymakers with a structured blueprint to design interventions that are culturally relevant, behaviorally informed, and scalable across diverse Arab countries. (Figure 3).

The Arab Context Nudge Model (ACNM)

A three-layer model integrating:

Layer 1: COM-B Core (Behavioral Mechanisms)

- Capability
- Opportunity
- Motivation

Layer 2: Arab Cultural Determinants

These shape how children and families make food decisions:

1. Family Hierarchy & Collectivism: caregivers influence children’s food choices
2. Religious Values: moderation, avoiding waste, fasting, discipline
3. Gender Norms: girls’ physical activity is significantly lower
4. Hospitality Culture: overeating encouraged
5. Food Symbolism: sweet foods associated with celebration and affection
6. Digital & Social Media Influence: high screen time, targeted food advertising
7. School as Cultural Influence → peer influence is very strong

Layer 3: Nudge Points

Where nudges can be applied realistically:

a. Home Environment

- Healthy defaults (fruits visible, sugary snacks less accessible)
- Plate size reduction
- Culturally tailored portion guidance based on traditional dishes
- Religious framing (e.g., moderation as “wasatiyya”)

b. Schools

- Rearranging canteens to make fruits/vegetables most accessible
- Using social norm messages (“Most students choose water today”)
- Gamified physical activity challenges
- Traffic-light labelling for traditional foods

c. Community and Digital Spaces

- Influencer-led social norm nudges
- Geo-targeted reminders around schools (“Try a healthy snack today”)
- Religious and cultural framing during Ramadan & celebrations
- Mobile apps for parents with nudges and tips.

### Ethical considerations

Designing nudges within public health policy, including childhood obesity interventions, requires careful ethical examination. While the principle of “libertarian paternalism” underpins nudging, guiding behavior without restricting choice, it has faced criticism for its potential misuse. Policymakers could leverage behavioral insights in ways that benefit certain stakeholders rather than the majority, creating what is referred to as “sludge.” Sludge uses cognitive biases and choice architecture similarly to nudges, but instead of facilitating healthier decisions, it introduces friction, complicating informed choice and potentially undermining public welfare. For example, a school policy might encourage children to “choose healthier snacks” but only provide expensive packaged “healthy” products in the cafeteria, rather than affordable fresh fruits or culturally familiar options. In this case, the policy could unintentionally benefit certain vendors or brands without genuinely improving children’s nutrition or promoting equitable access to healthy foods. To ensure that nudges promote ethical and equitable outcomes, both policymakers and communities must actively monitor, evaluate, and safeguard interventions so that nudges genuinely enhance the health and well-being of children and families, respecting autonomy while fostering positive behavior change (11).

### Study limitations

This scoping review presents various methodological limitations that need to be recognized. Initially, the focus on English-language publications might have created a language bias, possibly omitting pertinent studies released in Arabic or other languages. Secondly, publication bias might exist since studies showcasing positive results are more prone to being published and available. Third, even though the review included grey literature, it is possible that some relevant policy documents or program reports from Arab nations were not included. Fourth, the review depends solely on secondary data; no primary data were gathered from children, families, or healthcare providers in the area. Ultimately, the evaluation of the cultural relevance of interventions and behavioral insights relied on the authors’ interpretation instead of established cultural adaptation or validation procedures.

These constraints highlight the importance of future studies to enhance proof and relevance in the Arab setting. Empirical research examining nudges, the feasibility of interventions, and their effectiveness in home, school, and community environments is crucial. Moreover, consulting with local health experts, educators, and policymakers can enhance the design of interventions that are culturally sensitive. Future research must assess long-term behavioral effects and incorporate participatory methods that engage families and children in collaboratively designing interventions. In spite of these constraints, this review offers a foundational synthesis that aids in the creation of the Arab Context Nudge Model (ACNM) and directs evidence-driven, culturally appropriate childhood obesity prevention strategies in the area.

## Conclusion

Childhood obesity in Arab communities is considered a multifaceted challenge that requires a comprehensive, culturally sensitive approach. The most effective interventions are typically tailored to local contexts, considering existing social, environmental, and cultural factors. Actively involving and empowering local stakeholders, children, and families within communities is crucial for fostering sustainable, inclusive, and equitable changes in lifestyle and behavior. This study aimed to examine the behavioral origins of childhood obesity in Arab populations, analyse the implementation of behavioral interventions around the world, and identify useful insights for local policy initiatives. Leveraging behavioral insights and adopting nudging strategies can create sustainable changes in dietary behaviors. In the Arabic health policy landscape, interventions remain heavily focused on regulatory and tax-based measures, such sugar tax. Meanwhile, health-promoting subsidies remain limited, and nudging strategies are still underutilized within policy implementation. However, it is important to acknowledge that the effectiveness of nudging interventions is influenced by multiple contextual factors. Further research is required to clarify the specific mechanisms and conditions that enable nudges to work effectively. Future studies should focus on identifying these mechanisms in greater detail, as well as examining the long-term impact and sustainability of such interventions. Ultimately, childhood obesity is not just a medical issue, it is a behavioral, environmental, and cultural challenge. Tackling it through informed, evidence-based nudges rooted in the daily realities of Arab children and their families is essential to nurturing a healthier generation.
