# Correction: Policy paper: “Nudging for better nutrition: behavioral strategies to address childhood obesity in Arab communities” policy recommendations for action

**DOI:** 10.3389/fpubh.2026.1828303

**Published:** 2026-03-17

**Authors:** Frontiers Production Office

**Affiliations:** Frontiers Media SA, Lausanne, Switzerland

**Keywords:** childhood obesity, public health interventions, Arab countries, policy design, behavioral change, nudge

Author Immanuel Azaad Moonesar was erroneously assigned to affiliation 2 “School Health Physician, Sharjah Public School, Sharjah, United Arab Emirates”. This affiliation has now been removed for author Immanuel Azaad Moonesar. Author Immanuel Azaad Moonesar should be affiliated to affiliation 1 “Department of Academic Affairs (Health Policy), Mohammed Bin Rashid School of Government, Dubai, United Arab Emirates”.

The original version of this article has been updated.

